# Brief Adaptation to Astigmatism Reduces Meridional Anisotropy in Contrast Sensitivity

**DOI:** 10.1167/iovs.64.12.4

**Published:** 2023-09-01

**Authors:** Tsz-Wing Leung, Roger W. Li, Chea-Su Kee

**Affiliations:** 1 School of Optometry, Hong Kong Polytechnic University, Hung Hom, Kowloon, Hong Kong SAR; 2 Research Centre for SHARP Vision, Hong Kong Polytechnic University, Hung Hom, Kowloon, Hong Kong SAR; 3 Centre for Eye and Vision Research (CEVR), 17W Hong Kong Science Park, Hong Kong; 4 College of Optometry, Nova Southeastern University, Fort Lauderdale, Florida, United States

**Keywords:** astigmatism, directional blur, neural adaptation, meridional contrast sensitivity, meridional anisotropy

## Abstract

**Purpose:**

To investigate the effect of visual adaptation to orientation-dependent optical blur on meridional contrast sensitivity function in artificially imposed astigmatism.

**Methods:**

The study adopted a top-up adapt-test paradigm. During the blur adaptation process, the 18 non-astigmatic young adult participants were briefly presented with natural scene images (first trial, 10 minutes; subsequent trials, 6 seconds). Contrast sensitivities for horizontal and vertical gratings at spatial frequencies ranging from 1 to 8 cycles per degree (cpd) were measured immediately before and after adaptation to +3.00 diopters cylinder (DC) with-the-rule or against-the-rule astigmatism. Meridional anisotropy was measured to quantify the contrast sensitivity difference between the two grating orientations.

**Results:**

Adapting to astigmatic blur enhanced contrast sensitivity at the blurred power meridian but reduced contrast sensitivity at the least affected axis meridian. In with-the-rule conditions, contrast sensitivity for horizontal gratings was significantly increased, whereas that for vertical gratings was significantly decreased. Similarly, in against-the-rule conditions, contrast sensitivity for vertical gratings was significantly increased, whereas that for horizontal gratings was significantly decreased. These two factors together resulted in a substantial systematic reduction, averaging 34%, in meridional anisotropy of contrast sensitivity across the spatial frequency spectrum.

**Conclusions:**

Astigmatism adaptation occurs in natural scene viewing. Brief exposure to astigmatic blur altered contrast sensitivity in the opposite direction at the two principal meridians, indicating that the mature visual system possesses functional plasticity to recalibrate the response characteristics of orientationally tuned cortical filters and thus promote substantial reductions of meridional anisotropy in astigmatic vision, to some extent counterbalancing the elongated oval shape of astigmatic blur.

Astigmatism is an optical imperfection resulting from the difference in optical power between two perpendicular principal meridians. Approximately 20%^1^^–^^3^ to 40%^4^^,^^5^ of the population have at least 1 diopter (D) of refractive astigmatism. One distinct optical characteristic is that, unlike spherical refractive error producing non-directional blur, astigmatic refractive error produces orientation-dependent directional blur.^6^ When a beam of light passes through an astigmatic surface, two perpendicular image foci are formed at separate image planes along the optical axis. Unless at the circle of least confusion (i.e., the dioptric midway of the two image foci) at which there is non-directional blur, astigmatism degrades the retinal image quality for one orientation more than others, leading to permanent meridional visual deficits if not corrected early in life.^7^ Even in the absence of astigmatism in the central visual field, there is still remarkable off-axis astigmatism in the peripheral visual field of human eyes that increases dramatically with retinal eccentricity (eccentricity 30°: ∼3 D; eccentricity 60°: ∼9 D^8^^,^^9^). Spectacle lenses, particularly progressive addition lenses for correcting presbyopia, also produce unwanted astigmatism away from the optical center, due to the large amounts of astigmatism in the periphery on both sides of the central progressive zone of the lens.^10^

It is well known that optical defocus degrades the luminance contrast of retinal image contents, particularly at mid- and high spatial frequencies.^11^ Interestingly, there is evidence showing that adaptation to blur can modify visual functions.^12^ It is important that the testing procedures are not too lengthy; otherwise, most of the adaptation effect may have already decayed during the measurement. Surprisingly, later studies, using improved measurement strategies, demonstrated that contrast sensitivity was enhanced at a wide range of spatial frequencies following a brief period of adaptation to spherical defocus.^13^^,^^14^ Rajeev and Metha,^13^ using a top-up adaptation technique^15^^,^^16^ that consisted of a longer initial adaptation to spherical defocus, reported that in each subsequent testing trial the initially adapted images were briefly presented again for topping up the visual effect of blur adaptation. Although contrast sensitivity was found to be decreased at very low spatial frequency (0.5 cpd), adaptation to blur boosted contrast sensitivity at mid-spatial frequencies (8 and 12 cycles per degree [cpd]). Similar results were reported by Venkataraman et al.,^14^ who applied an efficient adaptive quick contrast sensitivity function technique, which allowed the entire contrast sensitivity function to be obtained in 3 minutes,^17^ and they demonstrated that blur adaptation enhanced contrast sensitivity at low spatial frequencies (3–4 cpd).

The majority of previous adaptation studies focused on spherical defocus that blurred the retinal image equally across orientations. However, a related study revealed that adaptation to digitally simulated astigmatic blur can alter visual perception and induce a strong orientation bias in the subsequently viewed images,^18^ thereby raising an interesting question of how the visual brain reacts when exposed to imposed astigmatism. In individuals with simple myopic astigmatism, with-the-rule (WTR) astigmatism diffuses the light in a vertical direction and blurs the horizontal contours, whereas against-the-rule (ATR) astigmatism diffuses in a horizontal direction instead and blurs the vertical contours. As astigmatism blurs the retinal image unequally across orientations, visual adaptation to directional blur in astigmatic defocus may adapt differently than non-directional blur in spherical defocus. We wondered whether orientation tuning can affect how the visual system responds when adapting to directional blur.

The experiments aimed to determine whether optically induced meridional anisotropy of contrast sensitivity could be, to a certain extent, compensated by neural adaptation to directional blur in astigmatism. A top-up adapt-test paradigm similar to that of previous studies^13^^,^^15^^,^^16^ was employed to characterize the orientation specificity of contrast sensitivity adaptation to astigmatic blur. Specifically, the transient adaptational response of the meridional contrast sensitivity function to artificially imposed astigmatism was measured along the two principal meridians.

## Methods

### Participants

Eighteen visually healthy young adults (18–35 years old; mean ± SE, 20.0 ± 0.30), all having ≤0.50 diopter cylinder (DC) astigmatism, participated. The spherical equivalent error ranged from plano to −5.00 D (mean ± SE, −2.11 ± 0.51). Hyperopia was not included, in order to avoid confounding ocular accommodation, which could potentially cause unpredictable changes in the directional blur pattern induced by astigmatic defocus.^6^ Refractive errors were measured by non-cycloplegia subjective refractions using the criterion of maximum plus for maximum visual acuity.^19^ Exclusion criteria included amblyopia, strabismus, history of ocular pathology and surgeries, anisometropia of 2.00 D or more, and best-corrected visual acuity worse than logMAR −0.1.

The experimental procedures were approved by the human ethics committee of The Hong Kong Polytechnic University (HSEARS20220106001), and the research was conducted according to the tenets of the Declaration of Helsinki. The experiments were undertaken with the understanding and written informed consent of each participant.

### Visual Stimuli

A psychophysical contrast sensitivity test was developed based on Psykinematix (KyberVision, Miyagi, Japan). A sinusoidal grating pattern (radius: 1.8°) was displayed in the center of the gamma-corrected liquid-crystal display (LCD) monitor screen (resolution: 1920 × 1080, refresh rate: 120 Hz, 10-bit RGB; Display++; Cambridge Research System, Rochester, UK). The background luminance was 50 cd/m^2^. To minimize any abrupt contrast cues, the grating edge was smoothed by a half-Gaussian ramp (σ = 0.2°) and the onset and offset of gratings by a temporal Gaussian envelope (σ = 50 ms). In each trial, a horizontal or vertical grating pattern was displayed for 500 ms. The observer’s task was to identify the grating orientation, horizontal or vertical, using a keyboard. No audio feedback to the observer's response was provided.

### Psychophysical Measurement

The contrast thresholds for horizontal and vertical gratings were measured at four spatial frequencies (1, 2, 4, and 8 cpd). To minimize visual fatigue caused by prolonged measurement procedures, only two spatial frequencies were tested in each run of approximately 30 to 45 minutes. The measurement was repeated until each spatial frequency was tested three times. The testing sequence of grating spatial frequencies was randomized without replacement:

• 1 and 2 cpd
• 1 and 4 cpd
• 1 and 8 cpd
• 2 and 4 cpd
• 2 and 8 cpd
• 4 and 8 cpd

Four randomly interleaved staircases were implemented for two grating orientations (horizontal and vertical gratings) and two spatial frequencies. Each staircase consisted of 60 trials, totaling 240 trials for each run. The grating contrast was controlled by the Ψ Bayesian adaptive algorithm.^20^ Contrast threshold was defined as the percent contrast at the 75% correct response rate, obtained by fitting a Weibull psychometric function. The contrast sensitivity reported was the reciprocal of mean contrast threshold based on three measurements.

### Baseline Contrast Sensitivity Measurements

The contrast sensitivity measurements were first performed with full optical correction using trial lenses. A +3.00 DC cylindrical lens was then added over the distance prescription with the axis placed at 180° and 90° to impose simple myopic WTR and ATR astigmatism, respectively. Pre-adaptation contrast sensitivity with astigmatism was measured immediately after cylindrical lens insertion. The testing sequence for WTR and ATR astigmatism was randomized. The monitor screen was viewed monocularly at 5 meters. Only the right eye was tested, the left eye being covered by a standard opaque eye patch. The experiment was conducted in a dim room. A 4-mm artificial pupil was placed 8 mm in front of the cornea to control for retinal illuminance.

### Adaptation to Astigmatic Defocus

**Figure 1.**
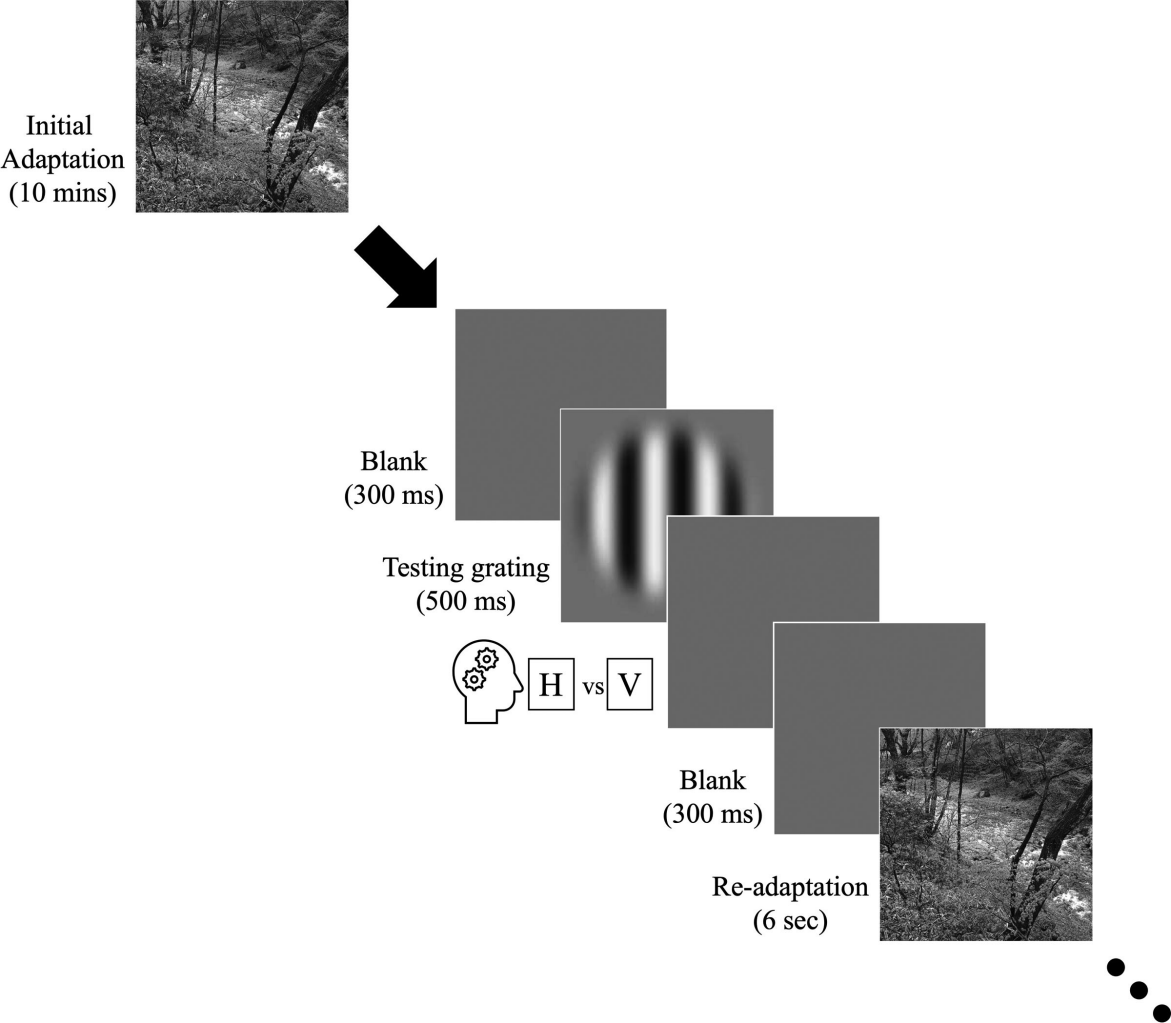
Top-up blur adaptation paradigm. With-the-rule (WTR) and against-the-rule (ATR) astigmatism was artificially induced by imposing a +3 D cylindrical spectacle lens with the axis of astigmatism placed at 180° or 90°, respectively. The adaptation protocol began with a 10-minute exposure to astigmatically blurred natural scene images, during which a set of 16 black-and-white photographs was randomly displayed every 500 ms. Immediately after the initial adaptation, a blank gray screen was briefly displayed for 300 ms, followed by a target sine-wave grating pattern for 500 ms. The observers’ visual task was to determine the grating orientation. Starting from the second trial, the presentation sequence returned to a shorter 6-second duration for top-up readaptation. Measurements were repeated until the contrast thresholds for all four spatial frequencies (1, 2, 4, and 8 cpd) and two grating orientations (horizontal and vertical) were obtained.

It has been demonstrated that brief exposure to astigmatic blur for 10 minutes alters visual sensitivity.^21^ To allow sufficient time to adapt, a similar blur adaptation period was adopted (Fig. 1). In the first trial, observers were required to adapt to astigmatic blur for 10 minutes. Sixteen black-and-white natural scene photos (size: 4.4° × 4.4°, pixels: 540 × 540) were then displayed in the center of the monitor screen, a new photograph being randomly selected every 500 ms to minimize light adaptation to local luminance profiles.

Because the visual adaptation effect can dissipate quickly after removal of the adapting stimulus,^22^ a top-up readaptation design as in other related visual adaptation studies^23^ was applied. Following adaptation, a blank gray field was displayed for 300 ms, followed by the testing grating stimulus for another 500 ms. The visual task was to identify the grating orientation identical to the baseline contrast sensitivity measurement procedures. In subsequent trials, the readaptation duration was reduced to 6 seconds. The measurement procedures were repeated until the contrast thresholds for four spatial frequencies and two grating orientations were obtained (240 trials each run; total: 240 trials × 6 runs = 1440 trials for each of the two astigmatism conditions).

### Meridional Anisotropy in Contrast Sensitivity

Meridional anisotropy is defined as the logarithmic difference in contrast sensitivity between the horizontal (*CS_H_*) and vertical (*CS_V_*) grating orientations:

$$\begin{array}{c} Meridional\;anisotropy=\log\left(CS_{H}\right)-\log\left(CS_{V}\right) \end{array}$$

Positive meridional anisotropy represents higher contrast sensitivity for horizontal gratings than vertical gratings, whereas negative meridional anisotropy represents lower contrast sensitivity for horizontal gratings than vertical gratings.

## Results

Of the 18 young adults who participated in this study, 17 completed the WTR astigmatism adaptation condition and 16 completed the ATR astigmatism adaptation condition. The remaining three participants completed only one astigmatism condition because of the city lockdown during the COVID-19 outbreak, which prevented attendance at the laboratory.

### Contrast Sensitivity With Full Optical Correction

**Figure 2.**
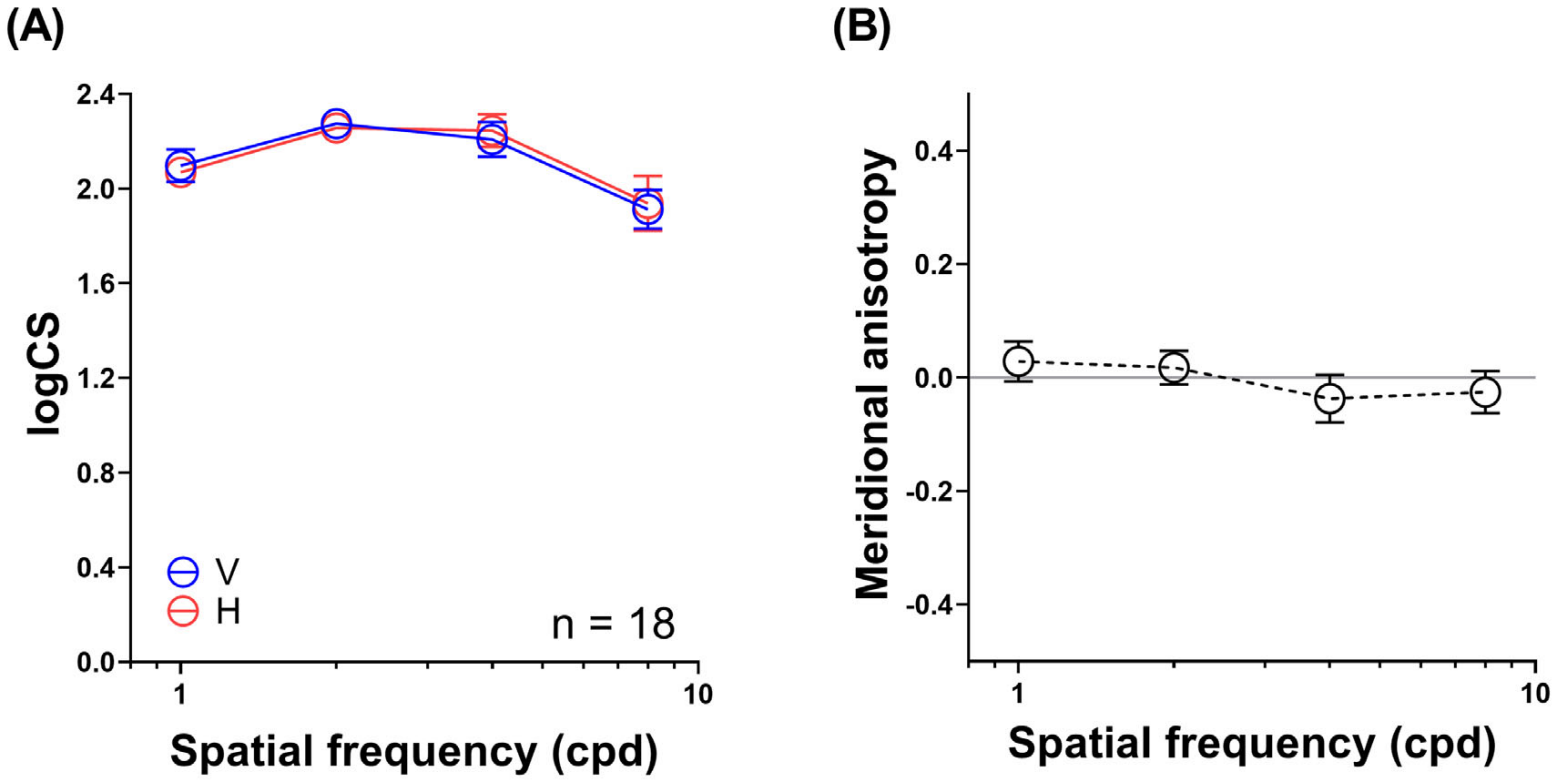
Contrast sensitivity and meridional anisotropy functions with full optical correction. (**A**) Contrast sensitivity function with full optical correction. With no artificially imposed astigmatism, there were no significant differences in contrast sensitivity between horizontal and vertical gratings (*red* and *blue symbols*, respectively) across the spatial frequencies tested. (**B**) Meridional anisotropy function in contrast sensitivity with full optical correction. No statistically significant meridional anisotropy in contrast sensitivity for the two grating orientations was observed across spatial frequencies. *Error bars:* 1 SEM.

With full optical correction, the participants had comparable contrast sensitivity for both horizontal and vertical gratings across all spatial frequencies tested: two-factor ANOVA repeated measure, orientation (horizontal and vertical gratings): *F*(1, 17) = 0.05, *P* = 0.83; orientation × spatial frequency (1, 2, 4, and 8 cpd) interaction: *F*(3, 51) = 0.82, *P* = 0.49 (Fig. 2). As a result, no remarkable meridional anisotropy in sensitivity for the two grating orientations was observed; that is, meridional anisotropy was not significantly different from zero for all spatial frequencies tested (one-sample *t*-tests, *t =* −0.89 to 0.81; *P ≥* 0.39).

### Pre-Adaptation Contrast Sensitivity With Imposed Astigmatism

**Figure 3.**
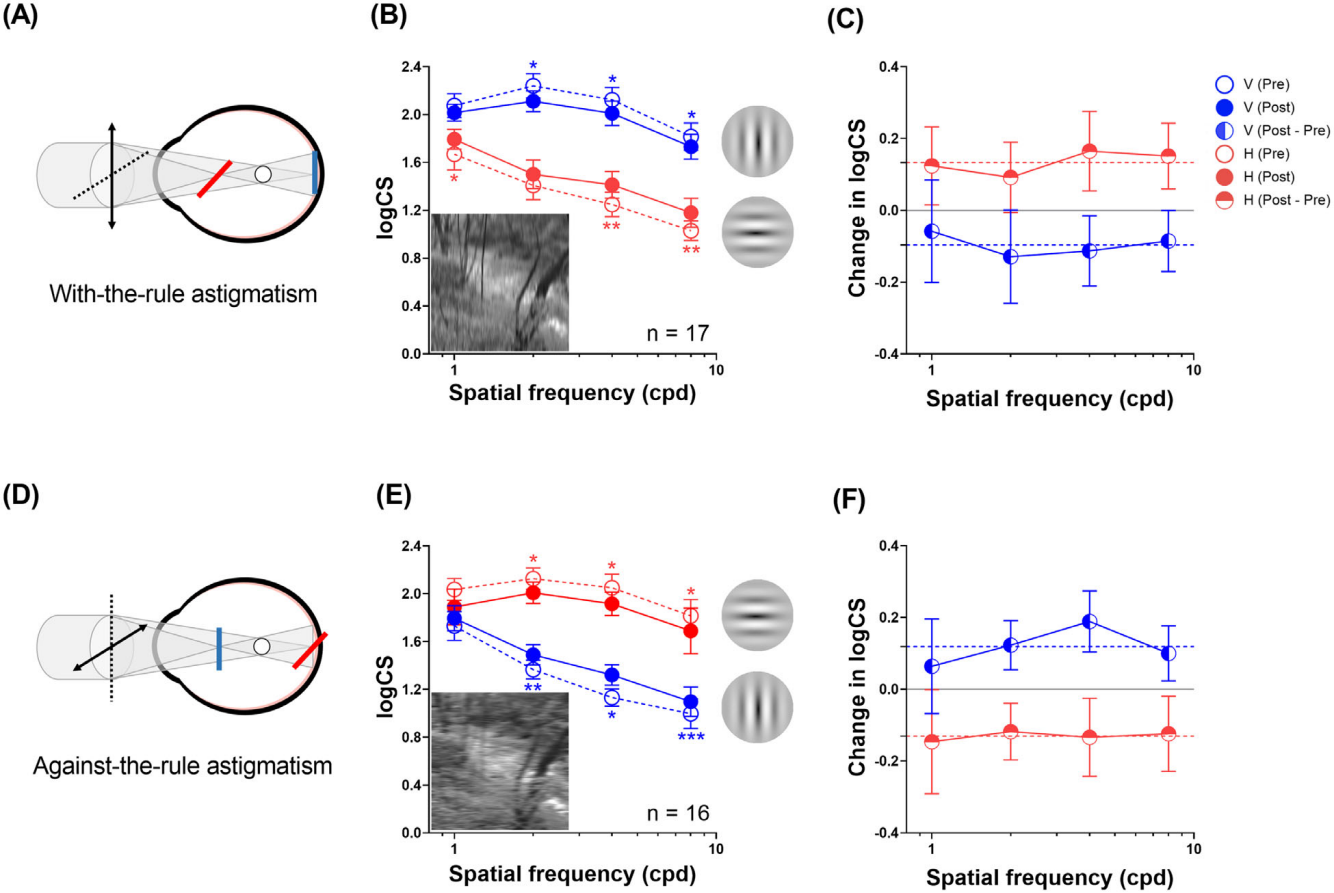
Neural adaptation to directional optical blur in WTR and ATR astigmatism (upper and lower row, respectively). (**A**, **D**, *left panels*) Directional astigmatic defocus produced by a +3 DC cylindrical lens. *Solid black lines* indicate principal power meridians with +3 D refractive power. *Dotted black lines* indicate principal axis meridians with zero refractive power. WTR astigmatism smeared retinal images vertically, but ATR astigmatism smeared retinal images horizontally (*blue* and *red lines*, respectively). (**B**, **E**, *middle panels*) Log contrast sensitivity for horizontal and vertical gratings (*red* and *blue symbols*, respectively) as a function of spatial frequency before and after astigmatic adaptation (*open symbols*, pre-adaptation; *closed symbols*, post-adaptation). Note that the pre-adaptation contrast sensitivity function for horizontal gratings was substantially decreased by WTR astigmatism, especially at spatial frequencies of 2 to 8 cpd (*top panel*, *red dashed line*) and that, similarly, the pre-adaptation contrast sensitivity function for vertical gratings was substantially decreased by ATR astigmatism (*bottom panel*, *blue dashed line*)*. Insets:* Simulated natural scene images illustrating directional blur produced by WTR and ATR astigmatism, blurred vertically and horizontally, respectively. Simple main effects analyses (post- vs. pre-): *P < 0.05, **P < 0.01, ***P < 0.001. (**C**, **F**, *right panels*) Magnitude of adaptation effect is the difference in log contrast sensitivity before and after astigmatic adaptation (logCS_Post_ – logCS_Pre_) as a function of spatial frequency. *Dashed lines* indicate the mean adaptation effect across spatial frequencies. *Positive values* represent enhancement in contrast sensitivity; *negative values*, reduction in contrast sensitivity. *Error bars:* 1 SEM.

As illustrated in the inset figures, simple myopic WTR astigmatism blurs the retinal image vertically, and simple myopic ATR astigmatism blurs the image horizontally (Figs. 3A, 3D). Imposing artificial astigmatism impacted (baseline, pre-adaptation) the contrast sensitivity function for horizontal and vertical gratings to a different extent depending on the axis of astigmatism.

As expected, WTR astigmatism substantially reduced contrast sensitivity for horizontal gratings but not vertical gratings across spatial frequencies: two-factor ANOVA repeated measures, orientation × spatial frequencies interaction: *F*(3, 48) = 14.99, *P* < 0.001; Bonferroni's post hoc tests: *t* ≤ −5.57 (all *p* < 0.001) (Fig. 3B, dashed lines, open symbols). In contrast, ATR astigmatism substantially reduced contrast sensitivity for vertical gratings but not horizontal gratings across spatial frequencies: two-factor ANOVA repeated measures, orientation × spatial frequency interaction: *F*(3, 45) = 32.98, *P* < 0.001; Bonferroni's post hoc tests: *t* ≤ −5.25 (all *p* < 0.001) (Fig. 3E, dashed lines, open symbols).

### Pre-Adaptation Meridional Anisotropy With Imposed Astigmatism

**Figure 4.**
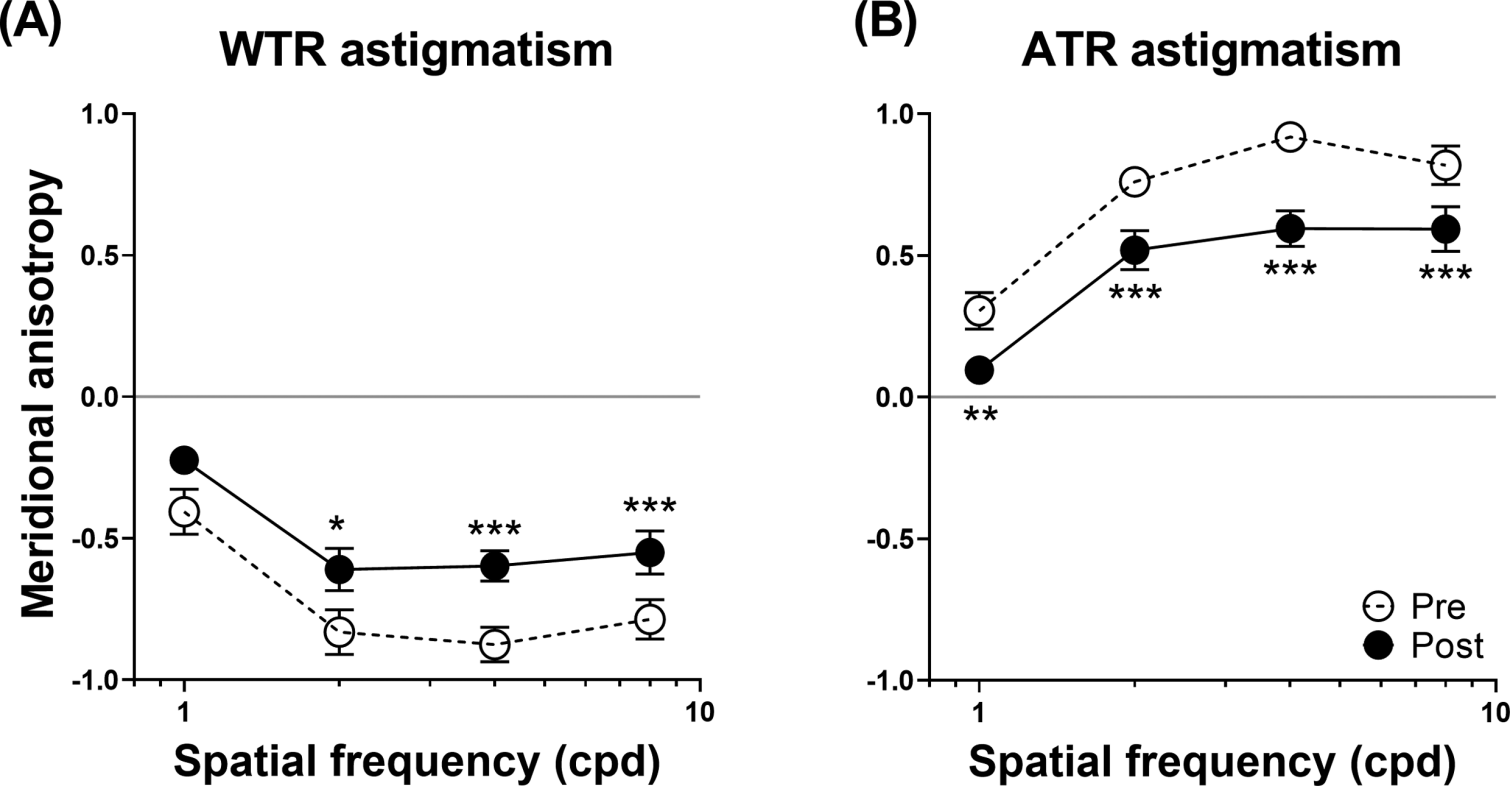
Adaptation-related reduction of meridional anisotropy in astigmatic vision. Meridional anisotropy in contrast sensitivity before and after adaptation to WTR (**A**) and ATR (**B**) astigmatism. Adaptation to astigmatism caused a systematic reduction of meridional anisotropy (i.e., toward the value of zero; *CS_H_* = *CS_V_*) across spatial frequencies. Meridional anisotropy was defined as the logarithmic difference in contrast sensitivity for horizontal and vertical gratings before (*dashed lines*, *open symbols*) and after (*solid lines*, *closed symbols*) adaptation. Positive meridional anisotropy indicates higher contrast sensitivity for horizontal gratings compared with vertical gratings; negative meridional anisotropy, lower contrast sensitivity for horizontal gratings compared with vertical gratings. Simple main effects analyses (post- vs. pre-): *P < 0.05, **P < 0.01, ***P < 0.001. *Error bars:* 1 SEM.

Notably, astigmatism induced different amounts of meridional anisotropy (baseline, pre-adaptation) in contrast sensitivity across the spatial frequencies tested. In WTR astigmatism, meridional anisotropy was found to be significantly more profound (i.e., more negative) at higher spatial frequencies than lower spatial frequencies: one-factor ANOVA repeated measures: *F*(4, 48) = 14.99, *P* < 0.001 (Fig. 4A, dashed lines, open symbols). Meridional anisotropy at 1 cpd was significantly less negative than that at higher spatial frequencies (Bonferroni's post hoc tests: *t* ≥ 4.83, all *P* < 0.001); no significant difference in meridional anisotropy was observed among 2, 4, and 8 cpd (Bonferroni's post hoc tests: *t* ≤ 0.56; all *P* > 0.79).

Similarly, in ATR astigmatism, a greater amount of meridional anisotropy (i.e., more positive) was found at higher spatial frequencies, when compared with lower spatial frequencies (1 cpd): one-factor ANOVA repeated measures: *F*(3, 45) = 32.98, *P* < 0.001; Bonferroni's post hoc tests: *t* ≤ −6.81, *P* < 0.001) (Fig. 4B, dashed lines, open symbols); the differences among higher spatial frequencies were not significant (Bonferroni's post hoc tests: *t* ≥ −2.36, *P* ≥ 0.07).

### Blur Adaptation to Imposed Astigmatism

Interestingly, it was observed that brief exposure to astigmatically blurred natural scene images remarkably altered contrast sensitivity at the two principal meridians in the “opposite” direction. Adaptation to artificially imposed astigmatism significantly and substantially boosted contrast sensitivity at the blurred meridian (WTR, horizontal gratings; ATR, vertical gratings). The entire contrast sensitivity function shifted upward, by 33% and 26% across spatial frequencies for WTR astigmatism (Fig. 3B, red open symbols versus red solid symbols) and ATR astigmatism (Fig. 3E, blue open symbols versus blue solid symbols), respectively; repeated measures two-factor ANOVA: WTR, *F*(1, 16) = 16.14, *P* < 0.001; ATR: *F*(1, 15) = 23.43, *P* < 0.001.

In contrast, contrast sensitivity at the orthogonal unblurred meridian significantly and remarkably declined. The entire contrast sensitivity function shifted downward by 22% and 25% across spatial frequencies for WTR astigmatism (Fig. 3B, blue open symbols vs. blue solid symbols) and ATR astigmatism (Fig. 3E, red open symbols versus blue solid symbols), respectively; repeated measures two-factor ANOVA: WTR, *F*(1, 16) = 5.82, *P* = 0.03; ATR, *F*(1, 15) = 12.71, *P* = 0.003. There were no significant interactions between astigmatism adaptation and spatial frequency (*F* ≤ 1.53, *P* ≥ 0.22). The raw contrast sensitivity data in Figures 3B and 3E are replotted in Figures 3C and 3F to quantify the magnitude of blur adaptation (i.e., the difference between pre- and post-adaptation contrast sensitivity).

For WTR astigmatism, contrast sensitivity for horizontal gratings was significantly increased across all (*F* ≥ 5.87; all *P* ≤ 0.03) (Fig. 3B, red lines and symbols) except the 2-cpd spatial frequency (*F* = 3.93; *P* = 0.07), and the contrast sensitivity for vertical gratings was decreased significantly across all (*F* ≥ 4.56; all *P* ≤ 0.048) (Fig. 3B, blue lines and symbols), except the 1-cpd spatial frequency (*F* = 0.76; *P* = 0.40). Similarly for ATR astigmatism, the contrast sensitivity for vertical gratings was significantly increased across all spatial frequencies (*F* ≥ 7.79; all *P* ≤ 0.01) (Fig. 3E, blue lines and symbols), except 1 cpd (*F* = 1.06; *P* = 0.32), and the contrast sensitivity for horizontal gratings significantly decreased across all spatial frequencies (*F* ≥ 4.62; all *P* ≤ 0.048) (Fig. 3E, red lines and symbols). Supplementary Figure S1 illustrates the effects of astigmatism adaptation on adjusted contrast sensitivity relative to the baseline contrast sensitivity under full optical correction.

### Adaptation-Related Reduction of Meridional Anisotropy

To summarize, brief adaptation to astigmatism caused a substantial reduction in meridional anisotropy in astigmatic perception. Across spatial frequencies, adaptation to astigmatic blur resulted in remarkable 32% and 36% decreases in meridional anisotropy in both the WTR, two-factor ANOVA repeated measures, *F*(1, 16) = 22.52, all *P* < 0.001 (Fig. 4A), and ATR astigmatism conditions, two-factor ANOVA repeated measures, *F*(1, 15) = 36.45, all *P* < 0.001 (Fig. 4B), respectively.

In both astigmatic settings, the entire meridional anisotropy function shifted toward the magnitude of zero anisotropy. There were no significant interactions between astigmatism adaptation and spatial frequency (*F* ≤ 1.33; *P* ≥ 0.28). Meridional anisotropy became less negative across spatial frequencies in WTR astigmatism (*F* ≥ 6.77; all *P* ≤ 0.02). Notably, the decrease in anisotropy at 1 cpd was only marginally significant (*F* = 4.28; *P* = 0.06). In contrast, the meridional anisotropy became less positive across spatial frequencies in ATR astigmatism (*F* ≥ 9.95; all *P* ≤ 0.007).

## Discussion

Visual adaptation modifies neuronal responses dynamically to interact with continuously changing visual scenes.^24^^–^^26^ In the current study, the orientation selectivity of contrast sensitivity adaptation to artificially imposed astigmatism was characterized along two principal meridians. The findings clearly suggest that brief adaptation to astigmatism selectively enhanced contrast sensitivity function at the blurred power meridian (mean improvement, 30% across both WTR and ATR settings), but reduced contrast sensitivity function at the less affected axis meridian (mean reduction, 24% across both WTR and ATR settings). Importantly, these two factors in turn caused a systematic reduction in meridional anisotropy function across the spatial frequency spectrum, to some extent counterbalancing the elongated oval shape of astigmatic blur. These fundamental observations may at least partially explain the visual benefits of astigmatism adaptation, such as enhancing visual acuity in uncorrected astigmatism (0.7 lines of improvement on a standard logMAR letter chart)^21^ and alleviating the perceptual shape distortion of astigmatic images,^18^ as reported previously.

Rather than the commonly employed simple visual stimuli, such as sinusoidal gratings, natural scene images were used in the adaptation. The present findings demonstrate that astigmatic blur adaptation can take place in natural scene views that are typically encountered in the real world. In the framework of Fourier analysis, natural scene images consist of a broad range of spatial frequency and orientation components. The amplitude of the power spectrum falls proportionally, with a log–log slope of approximately –1, as spatial frequency increases.^15^ Previous related work also used a similar natural scene strategy to study contrast adaptation, another type of visual adaptation, to unblurred images.^23^

Using digital filters implemented in an augmented reality device, another study examined how the visual system adapted to orientation-specific visual deprivation.^27^ A full filter was used to remove contrast energy for all spatial frequencies at the deprived orientation, and a narrow filter was used to remove contrast energy selectively at lower spatial frequencies (0.6–4 cpd). Similar to our observations for astigmatic blur adaptation, adaptation to visual deprivation minimized the contrast sensitivity difference between the deprived meridian and the non-deprived meridian at low spatial frequency (1 cpd), although higher spatial frequencies were not tested in their study. It is notable that in the full-filter condition, contrast sensitivities at the two meridians were both reduced after deprivation adaptation, with the non-deprived meridian being more affected, and that in the narrow-filter condition contrast sensitivity was improved at the deprived meridian but remained unchanged at the non-deprived meridian. However, these observations need to be interpreted cautiously, as the decreased contrast sensitivity observed under the full-filter condition could be simply the result of visual fatigue with prolonged wear, as long as 4 hours, of a heavy headmount display in an unnatural visual environment.^27^ In the current study, the measurements were divided into 12 short testing sessions, each consisting of only 30 to 45 minutes, in order to minimize this confounding factor.

We postulate that the adaptive modifications of contrast sensitivity to astigmatism observed may be mediated by contrast gain control mechanisms at the cortical level.^28^^–^^30^ To preserve perceptual constancy or minimize meridional anisotropy, the contrast gain of those spatial filters tuned to the blurred orientation might have been increased to enhance contrast sensitivity and conversely, the contrast gain of those tuned to the orthogonal unblurred orientation might have been decreased to reduce contrast sensitivity. This type of blur adaptation has also proven to be beneficial for compensation of spherical defocus. After minutes to hours of exposure to defocused images, optically degraded visual acuity can be enhanced through blur adaptation, by as much as 0.5 to 2.5 lines of improvement on a standard logMAR acuity chart.^12^^,^^31^^–^^35^ It has been shown that adaptation to a blurred image makes the subsequent unfiltered image appear sharper, whereas adaptation to a sharpened image makes the subsequent unfiltered image appear blurry.^36^

A single-interval binary-choice paradigm was adopted in the contrast sensitivity measurement in order to display sufficient grating cycles (i.e., at least three cycles) for low spatial frequencies. However, these psychophysical procedures might have introduced response bias^37^ in the measurement—bias favoring one orientation could improve thresholds for that orientation while making them worse for the alternative orientation. Therefore, in a supplementary experiment, we applied a criterion-free forced-choice method by using a spatial two-alternative arrangement where the upper or lower half of the monitor screen area contained a circular grating stimulus and the other half-area was blank (Supplementary Fig. S2, inset). Because of the monitor screen constraints, contrast sensitivity was measured only for 4-cpd gratings before and after adaptation to +3.00 D astigmatism. In general, the meridional adaptation effects observed were indeed very similar to the binary-choice datasets reported in Figure 3 (Supplementary Fig. S2, upper row, WTR; lower row, ATR); that is, adaptation to astigmatic blur increased contrast sensitivity of the blurred meridian (Supplementary Fig. S2, top rightmost panel, red line; bottom rightmost panel, blue line) and decreased contrast sensitivity of the unaffected orthogonal meridian (Supplementary Fig. S2, top rightmost panel, blue line; bottom rightmost panel, red line). These supplementary criterion-free data further support our conclusions.

Here we show that astigmatism adaptation occurs in natural scene viewing in visually normal young adults. Our findings characterized the orientation specificity of contrast sensitivity adaptation to artificially imposed astigmatic blur, providing the evidence that the mature visual system possesses functional plasticity to recalibrate the response characteristics of spatial filters, possibly at the cortical level, during adaptation to uncorrected astigmatism. Our study adopted a relatively short-term adaptation strategy and investigated the transient effect of blur adaptation at only two cardinal orientations for with-the-rule and against-the-rule astigmatism. In our laboratories, we are currently studying the transfer of blur adaptation across orientations for oblique astigmatism (also see Vinas et al.^38^). Further investigations may be needed to characterize the prolonged profile of astigmatism adaptation and to examine meridional blur adaptation in patients with irregular astigmatism such as keratoconus.^39^

## Supplementary Material

Supplement 1
