# Supplementary material for: Brief Adaptation to Astigmatism Reduces Meridional Anisotropy in Contrast Sensitivity
Source: Invest Ophthalmol Vis Sci. 2023 Sep 1;64(12):4. doi: 10.1167/iovs.64.12.4 (PMC10479241; doi:10.1167/iovs.64.12.4)
Supplement: Supplement 1 [file iovs-64-12-4_s001.pdf]

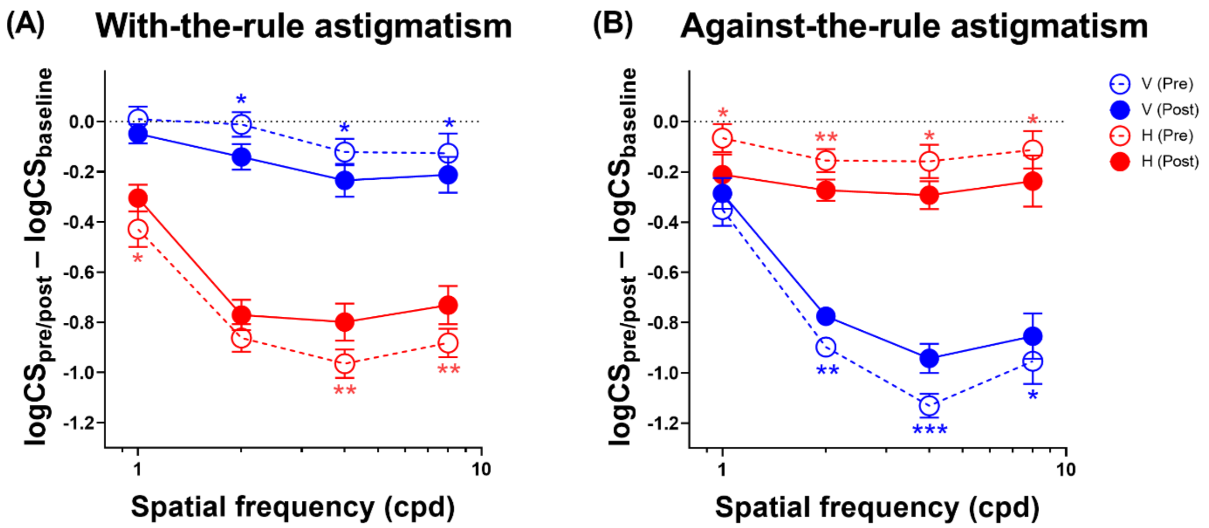

SUPPLEMENTARY FIGURE S1. Adaptation effects on adjusted contrast sensitivity, relative to the baseline condition with full optical correction. The calculations were based on the baseline contrast sensitivity data reported in Figure 2 - i.e., $logCS_{pre/post} – logCS_{baseline}$ for both horizontal (red symbols) and vertical gratings (blue symbols). Imposing WTR (**A**) and ATR (**B**) astigmatism resulted in a substantial reduction of contrast sensitivity for horizontal and vertical gratings (red and blue lines), respectively. Adaptation to astigmatism significantly increased adjusted contrast sensitivity at the blurred meridian (repeated measures two-factor ANOVA: WTR: $F(1, 16) = 16.14$, $p < 0.001$; ATR: $F(1, 15) = 23.43$, $p < 0.001$) and decreased contrast sensitivity at the orthogonal unblurred meridian ($F(1, 16) = 5.82$, $p = 0.03$; ATR: $F(1, 15) = 12.71$, $p = 0.003$). *Simple main effects analyses (Post vs Pre): * $p < 0.05$, ** $p < 0.01$, *** $p < 0.001$.* Upward shift, increase in sensitivity. Downward shift, decrease in sensitivity.

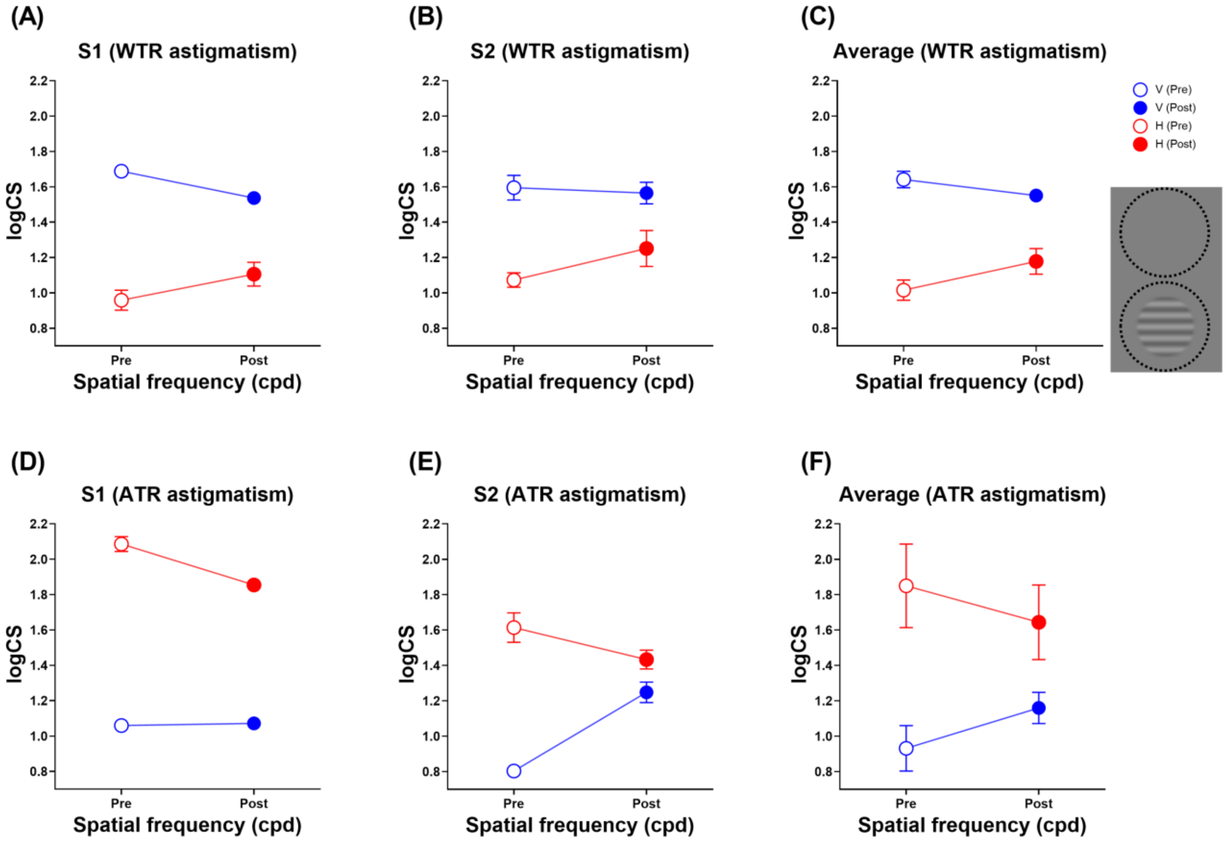

SUPPLEMENTARY FIGURE S2. Measurement of astigmatic blur adaptation using a two spatial alternative forced-choice method. The observers' task was to determine the grating location (inset, upper or lower). Two new adult observers with normal vision participated (S1, S2). Before and after adaptation to +3.00 D astigmatism, contrast sensitivity was measured for 4 cpd horizontal and vertical gratings. Grating stimulus parameters: stimulus radius, 0.6º; background luminance, 50 cd/m^2; stimulus duration, 500 ms; grating edge, smoothed by a half-Gaussian ramp, σ = 0.07º; onset and offset of gratings, smoothed by a temporal Gaussian envelope, σ = 50 ms. (**A** – **C**) WTR astigmatism. (**D** – **E**) ATR astigmatism. Similar to those data reported in Fig. 3 using a single-interval binary-choice paradigm, adaptation to astigmatism increased contrast sensitivity of the blurred meridian (top rightmost panel, red line; bottom rightmost panel, blue line) and decreased contrast sensitivity of the orthogonal unaffected meridian (top rightmost panel, blue line; bottom rightmost panel, red line). Open symbols, pre-adaptation. Closed symbols, post-adaptation.
